# Aberrantly methylated-differentially expressed genes and pathways in colorectal cancer

**DOI:** 10.1186/s12935-017-0444-4

**Published:** 2017-08-07

**Authors:** Jingwei Liu, Hao Li, Liping Sun, Zhenning Wang, Chengzhong Xing, Yuan Yuan

**Affiliations:** 1 Tumor Etiology and Screening Department of Cancer Institute and General Surgery, The First Affiliated Hospital of China Medical University, and Key Laboratory of Cancer Etiology and Prevention (China Medical University), Liaoning Provincial Education Department, Shenyang, 110001 China; 2 Tumor Etiology and Screening Department of Cancer Institute and General Surgery, The First Affiliated Hospital of China Medical University, 155# North Nanjing Street, Heping District, Shenyang, 110001 Liaoning China

**Keywords:** Methylation, Expression, Bioinformatics, Colorectal cancer

## Abstract

**Background:**

Methylation plays an important role in the etiology and pathogenesis of colorectal cancer (CRC). This study aimed to identify aberrantly methylated-differentially expressed genes (DEGs) and pathways in CRC by comprehensive bioinformatics analysis.

**Methods:**

Data of gene expression microarrays (GSE68468, GSE44076) and gene methylation microarrays (GSE29490, GSE17648) were downloaded from GEO database. Aberrantly methylated-DEGs were obtained by GEO2R. Functional and enrichment analyses of selected genes were performed using DAVID database. Protein–protein interaction (PPI) network was constructed by STRING and visualized in Cytoscape. MCODE was used for module analysis of the PPI network.

**Results:**

Totally 411 hypomethylation-high expression genes were identified, which were enriched in biological processes of response to wounding or inflammation, cell proliferation and adhesion. Pathway enrichment showed cytokine–cytokine receptor interaction, p53 signaling and cell cycle. The top 5 hub genes of PPI network were CAD, CCND1, ATM, RB1 and MET. Additionally, 239 hypermethylation-low expression genes were identified, which demonstrated enrichment in biological processes including cell–cell signaling, nerve impulse transmission, etc. Pathway analysis indicated enrichment in calcium signaling, maturity onset diabetes of the young, cell adhesion molecules, etc. The top 5 hub genes of PPI network were EGFR, ACTA1, SST, ESR1 and DNM2. After validation in TCGA database, most hub genes still remained significant.

**Conclusion:**

In summary, our study indicated possible aberrantly methylated-differentially expressed genes and pathways in CRC by bioinformatics analysis, which may provide novel insights for unraveling pathogenesis of CRC. Hub genes including CAD, CCND1, ATM, RB1, MET, EGFR, ACTA1, SST, ESR1 and DNM2 might serve as aberrantly methylation-based biomarkers for precise diagnosis and treatment of CRC in the future.

## Introduction

Colorectal cancer (CRC) is one of the most common malignant tumors with incidence ranked third in men and second in women worldwide [1]. Multiple factors might cause CRC including alcohol, smoking, obesity and insufficient physical exercise [2]. The accumulation of various genetic and epigenetic alternations in colorectal epithelial cells is also regarded as essential processes driving the initiation and progression of CRC [3].

Epigenetics is known as heritable alterations in gene expression that are not mediated by changes within the DNA sequence [4]. Cancer epigenetic covers aspects of DNA methylation, noncoding RNA and histone modification, of which the most widely investigated epigenetic changes is aberrant DNA methylation [5]. Aberrant methylation could influence the functions of key genes especially tumor suppressor genes through altering their expression, thus making it involved in various processes of CRC development [6]. Although multiple studies have demonstrated certain genes with aberrant DNA hypermethylation or hypomethylation in CRC, the comprehensive profile and pathways of the interaction network remain largely elusive.

In recent years, micoarrays based on high-throughput platforms emerge as a promising and efficient tool to screen significant genetic or epigenetic alternations in carcinogenesis and identify biomarkers for diagnosis and prognosis of cancer [7]. A number of gene expression profiling microarrays have been conducted to find various differentially expressed genes (DEGs) in CRC [8]. In addition, some studies concerning aberrant methylation in CRC have also been performed to indicate differentially methylated genes (DMGs) [9]. However, individual investigations possess limited numbers of overlapping gene profiling and insufficient power to identify key genes and pathways involved in multiple cellular process and biological function. Now, by the means of advanced bioinformatics analysis of available microarray data, it is possible to come up with more reliable and precise screening results via overlapping relevant data sets.

Until now, no research has been performed to jointly analyze information of both gene expression profiling microarray and gene methylation profiling microarray in the development of CRC. In the present study, data of gene expression profiling microarrays (GSE68468, GSE44076) and gene methylation profiling microarrays (GSE29490, GSE17648) were integrated and analyzed by a series of bioinformatics tools. Aberrantly methylated-DEGs and pathways were identified in CRC. Protein–protein interaction network was constructed and hub genes were revealed. By this means, we expect to find novel aberrantly methylated genes and pathways in CRC and shed light on the underlying molecular mechanisms that orchestrate colorectal carcinogenesis.

## Materials and methods

### Microarray data

In the present study, the gene expression profiling data sets (GSE68468, GSE44076) and gene methylation profiling data sets (GSE29490, GSE17648) were obtained from Gene Expression Omnibus (GEO, https://www.ncbi.nlm.nih.gov/geo/) of The National Center for Biotechnology Information (NCBI). Totally 186 CRC and 55 normal mucosa specimens were enrolled in GSE68468 (platform: GPL96 Affymetrix Human Genome U133A Array) while 98 CRC and 50 normal mucosa specimens were enrolled in GSE44076 (platform: GPL13667 Affymetrix Human Genome U219 Array). For gene methylation profiling microarray, GSE29490 included a total of 48 paired normal and CRC samples from 24 patients while GSE17648 included altogether 44 paired normal and CRC samples from 22 patients. Both of these two methylation microarray used platform GPL8490 (Illumina HumanMethylation27 BeadChip).

### Data processing

We used GEO2R online software to analysis the raw submitter-supplied data of microarrays and identify DEGs and DMGs. GEO2R is an interactive web tool which allows users to compare different groups of samples in a GEO series in order to screen genes that are differentially expressed across experimental conditions. P < 0.05 and |t| > 2 were used as the cut-off criteria to find DEGs and DMGs. Subsequently, MATCH function was adopted to identify overlapping DEGs in the two gene expression profiling data sets of GSE68468 and GSE44076, and overlapping DMGs in the two gene methylation profiling data sets of GSE29490 and GSE17648. Finally, hypomethylation-high expression genes were obtained by overlapping hypomethylation and up-regulated genes; hypermethylation-low expression genes were obtained by overlapping hypermethylation and down-regulated genes.

### Functional and pathway enrichment analysis

The Database for Annotation, Visualization and Integrated Discovery (DAVID, https://david.ncifcrf.gov/) was used to perform functional and pathway enrichment analysis. DAVID offers systematic and integrative functional annotation tools for investigators to unravel biological meaning behind large list of genes [10]. Gene ontology (GO) analysis including the cellular component, molecular function, and biological process [11] and Kyoto Encyclopedia of Genes and Genomes (KEGG) pathway enrichment analysis [12] were conducted for the selected hypomethylation-high expression genes and hypermethylation-low expression genes by DAVID. P < 0.05 was regarded as statistical significance.

### Protein–protein interaction (PPI) network construction and module analysis

The functional protein–protein interaction (PPI) analysis is essential to interpret the molecular mechanisms of key cellular activities in carcinogenesis. In this study, we used Search Tool for the Retrieval of Interacting Genes (STRING) database to construct PPI network of hypomethylation-high expression genes and hypermethylation-low expression genes, respectively. Interaction score of 0.4 was regarded as the cut-off criterion and the PPI was visualized. Subsequently, the Molecular Complex Detection (MCODE) in Cytoscape software was conducted to screen modules within PPI network with MCODE score >3 and number of nodes >4. Hub genes were selected with connection degree >10. The functional enrichment analysis of the genes in individual module was achieved by DAVID with a significance threshold of P < 0.05.

### Validation of the hub genes in TCGA database

The Cancer Genome Atlas (TCGA) database, a collaboration between the National Cancer Institute (NCI) and National Human Genome Research Institute (NHGRI), has generated comprehensive, multi-dimensional maps of the key genomic changes in various types of cancers. In order to confirm the results, hypomethylation-high expression hub genes and hypermethylation-low expression hub genes were then validated in another database TCGA.

## Results

### Identification of aberrantly methylated-differentially expressed genes in CRC

Data from each microarray were separately analyzed by online software GEO2R to screen DEGs or DMGs. Fdor DEGs of gene expression microarray, 3411 overlapping up-regulated genes (4736 in GSE68468, 7618 in GSE44076) and 2623 overlapping down-regulated genes (3630 in GSE68468, 9686 in GSE44076) were identified. For DMGs of gene methylation microarray, 1534 overlapping hypermethylation genes (2071 in GSE29490, 2269 in GSE17648) and 3011 overlapping hypomethylation genes (3786 in GSE29490, 4907 in GSE17648) were found.

**Fig. 1:**
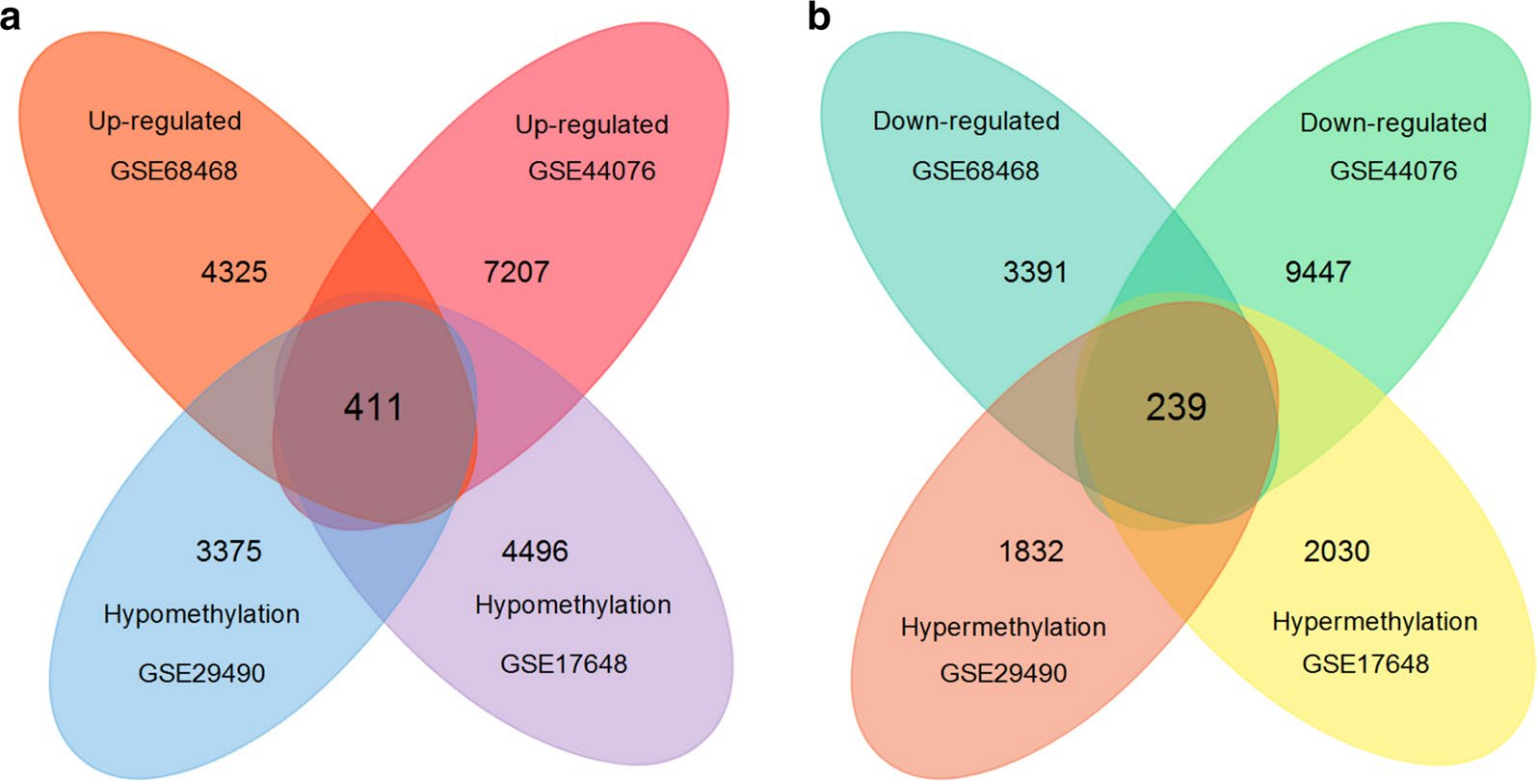
Identification of aberrantly methylated-differentially expressed genes in gene expression datasets (GSE68468, GSE44076) and gene methylation datasets (GSE29490, GSE17648) **a** hypomethylation and up-regulated genes; **b** hypermethylation and down-regulated genes

**Fig. 2:**
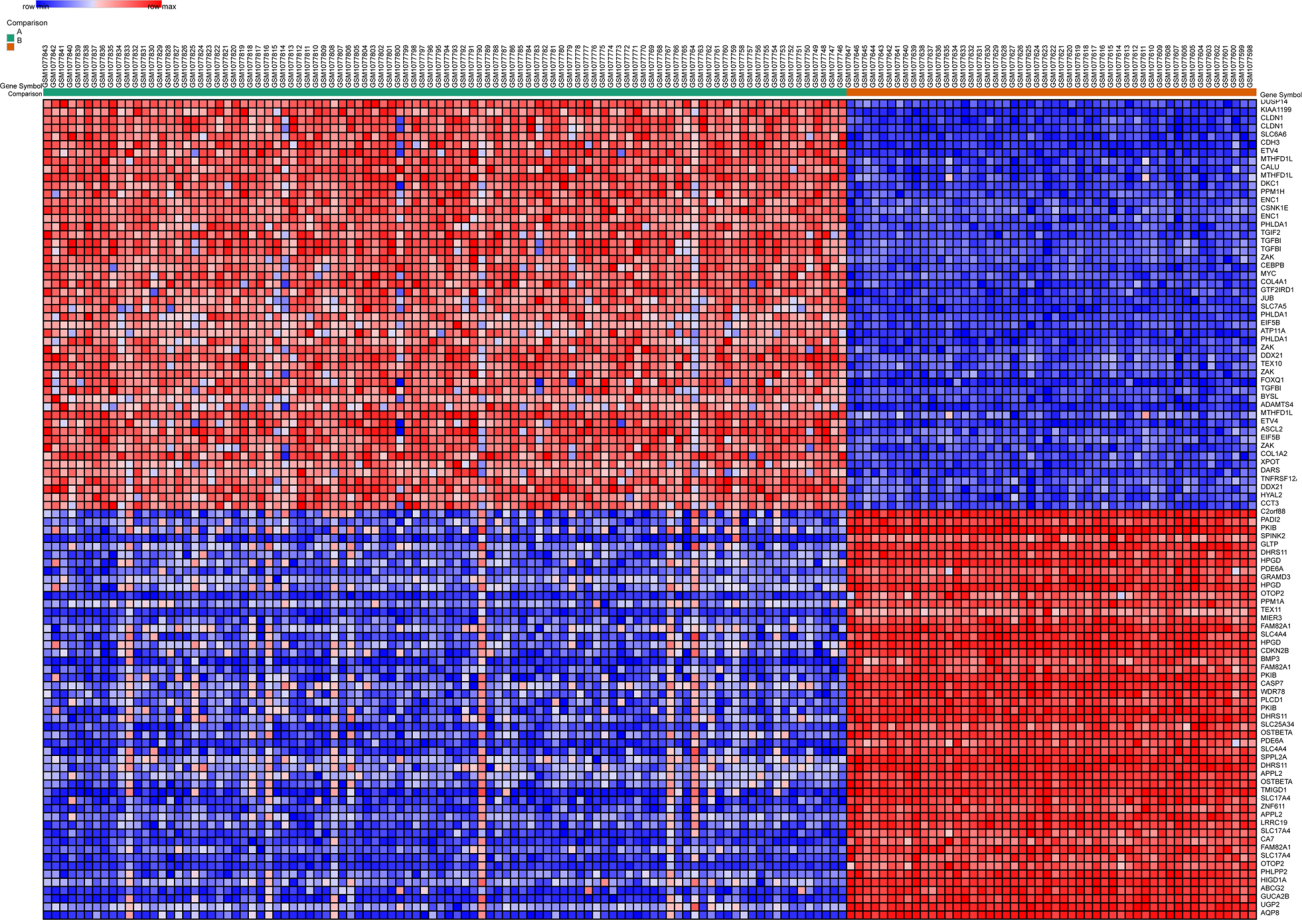
Representative heat map of the top 100 differentially expressed genes in dataset GSE44076 (50 up-regulated genes and 50 down-regulated genes). *Red* up-regulation; *blue* down-regulation

Then, totally 411 hypomethylation-high expression genes were obtained by overlapping 3011 hypomethylation genes and 3411 up-regulated genes; 239 hypermethylation-low expression genes were obtained by overlapping 1534 hypermethylation genes and 2623 down-regulated genes (Fig. 1). The representative heat map of GSE44076 (top 50 up-regulated and down-regulated genes) was shown in Fig. 2.

### GO functional enrichment analysis

The top 5 significant terms of GO enrichment analysis in DAVID were illustrated in Table 1. Hypomethylation-high expression genes were enriched in biological processes (BP) of response to wounding, inflammatory response, cell proliferation, cell adhesion and biological adhesion. As for molecular function (MF), these genes showed enrichment in identical protein binding, cytokine activity, endopeptidase activity, heparin binding, protein binding and bridging. Besides, cell component (CC) indicated enrichment predominantly at extracellular region, which indicated that hypomethylation-high expression genes might play a critical role in tumor microenvironment of CRC (Table 2).

**Table 1:** Gene ontology analysis of aberrantly methylated-differentially expressed genes in colorectal cancer

Category	GO analysis	Term	Gene count	%	P value
Hypomethylation and high expression					
	GOTERM_BP_FAT	GO:0009611~response to wounding	37	9.00	9.44E−08
	GOTERM_BP_FAT	GO:0006954~inflammatory response	25	6.08	3.48E−06
	GOTERM_BP_FAT	GO:0008283~cell proliferation	29	7.06	7.95E−06
	GOTERM_BP_FAT	GO:0007155~cell adhesion	37	9.00	5.54E−05
	GOTERM_BP_FAT	GO:0022610~biological adhesion	37	9.00	5.55E−05
	GOTERM_CC_FAT	GO:0005576~extracellular region	92	22.38	2.01E−08
	GOTERM_CC_FAT	GO:0044421~extracellular region part	55	13.38	3.78E−08
	GOTERM_CC_FAT	GO:0005615~extracellular space	42	10.22	4.12E−07
	GOTERM_CC_FAT	GO:0005578~proteinaceous extracellular matrix	21	5.11	2.45E−04
	GOTERM_CC_FAT	GO:0005625~soluble fraction	20	4.87	5.01E−04
	GOTERM_MF_FAT	GO:0042802~identical protein binding	35	8.52	8.25E−05
	GOTERM_MF_FAT	GO:0005125~cytokine activity	15	3.65	6.69E−04
	GOTERM_MF_FAT	GO:0004175~endopeptidase activity	21	5.11	2.33E−03
	GOTERM_MF_FAT	GO:0030674~protein binding, bridging	9	2.19	3.39E−03
	GOTERM_MF_FAT	GO:0008201~heparin binding	9	2.19	5.90E−03
Hypermethylation and low expression					
	GOTERM_BP_FAT	GO:0007267~cell–cell signaling	29	12.13	3.32E−07
	GOTERM_BP_FAT	GO:0019226~transmission of nerve impulse	18	7.53	4.70E−05
	GOTERM_BP_FAT	GO:0031644~regulation of neurological system process	11	4.60	1.72E−04
	GOTERM_BP_FAT	GO:0007610~behavior	20	8.37	1.90E−04
	GOTERM_BP_FAT	GO:0030182~neuron differentiation	19	7.95	2.35E−04
	GOTERM_CC_FAT	GO:0044459~plasma membrane part	64	26.78	2.13E−09
	GOTERM_CC_FAT	GO:0005886~plasma membrane	84	35.15	5.38E−07
	GOTERM_CC_FAT	GO:0043005~neuron projection	19	7.95	1.13E−06
	GOTERM_CC_FAT	GO:0005887~integral to plasma membrane	38	15.90	1.67E−06
	GOTERM_CC_FAT	GO:0031226~intrinsic to plasma membrane	38	15.90	2.85E−06
	GOTERM_MF_FAT	GO:0003700~transcription factor activity	38	15.90	2.48E−07
	GOTERM_MF_FAT	GO:0022836~gated channel activity	16	6.69	9.15E−05
	GOTERM_MF_FAT	GO:0015267~channel activity	18	7.53	2.17E−04
	GOTERM_MF_FAT	GO:0022803~passive transmembrane transporter activity	18	7.53	2.23E−04
	GOTERM_MF_FAT	GO:0005216~ion channel activity	17	7.11	3.17E−04

**Table 2:** KEGG pathway analysis of aberrantly methylated-differentially expressed genes in colorectal cancer

Pathway ID	Pathway name	Gene no.	%	P value	Genes
Hypomethylation and high expression					
hsa04060	Cytokine–cytokine receptor interaction	20	4.87	5.86E−04	CXCL1, IL2RA, LTBR, CXCL3, IL21R, CXCL2, MET, CXCL9, TNFSF15, TNFRSF4, CCL18, LIF, INHBA, TNFRSF9, TNFSF11, IL23A, PRLR, CCL20, IL20RA, ACVR1
hsa04115	p53 signaling pathway	8	1.95	5.54E−03	CCND1, SERPINB5, CD82, RRM2, TSC2, CASP8, PMAIP1, ATM
hsa04110	Cell cycle	9	2.19	0.045	CCND1, PKMYT1, PRKDC, RB1, CDC16, CDC27, ATM, WEE1, TFDP1
Hypermethylation and low expression					
hsa04020	Calcium signaling pathway	12	5.02	7.80E−04	EGFR, EDNRB, ADCY2, PTGER3, PDE1B, PDE1C, DRD5, GRIN2A, PLCD1, ITPKB, CAMK2B, PTGFR
hsa04950	Maturity onset diabetes of the young	5	2.09	1.45E−03	HHEX, NEUROD1, HNF4G, NEUROG3, NKX2-2
hsa04514	Cell adhesion molecules (CAMs)	8	3.35	0.017	NCAM1, CD8A, ITGA8, NLGN4X, CNTN1, ITGA4, JAM2, HLA-G
hsa04080	Neuroactive ligand-receptor interaction	11	4.60	0.033	EDNRB, SSTR2, PTGER3, GABRB3, GRIK1, GRIK2, DRD5, GRIN2A, GRIA3, PTGFR, VIPR2
hsa04062	Chemokine signaling pathway	9	3.77	0.035	DOCK2, ADCY2, CXCL14, TIAM1, HCK, PIK3CD, CCR10, RAP1A, ELMO1
hsa04666	Fc gamma R-mediated phagocytosis	6	2.51	0.043	DOCK2, HCK, PIK3CD, ARF6, AMPH, DNM2

For hypermethylation-low expression genes, enriched biological processes included cell–cell signaling, transmission of nerve impulse, regulation of neurological system process and neuron differentiation. Molecular function enrichment indicated transcription factor activity, gated channel activity, channel activity, passive transmembrane transporter activity and ion channel activity. Additionally, cell component displayed plasma membrane and neuron projection.

### KEGG pathway analysis

KEGG pathway enrichment analysis suggested that hypomethylation-high expression genes were significantly enriched in pathways including cytokine–cytokine receptor interaction, p53 signaling and cell cycle. Hypermethylation-low expression genes demonstrated enrichment in pathways of calcium signaling pathway, maturity onset diabetes of the young, cell adhesion molecules (CAMs), neuroactive ligand-receptor interaction, chemokine signaling and Fc gamma R-mediated phagocytosis.

### PPI network construction, module analysis and hub gene selection

**Fig. 3:**
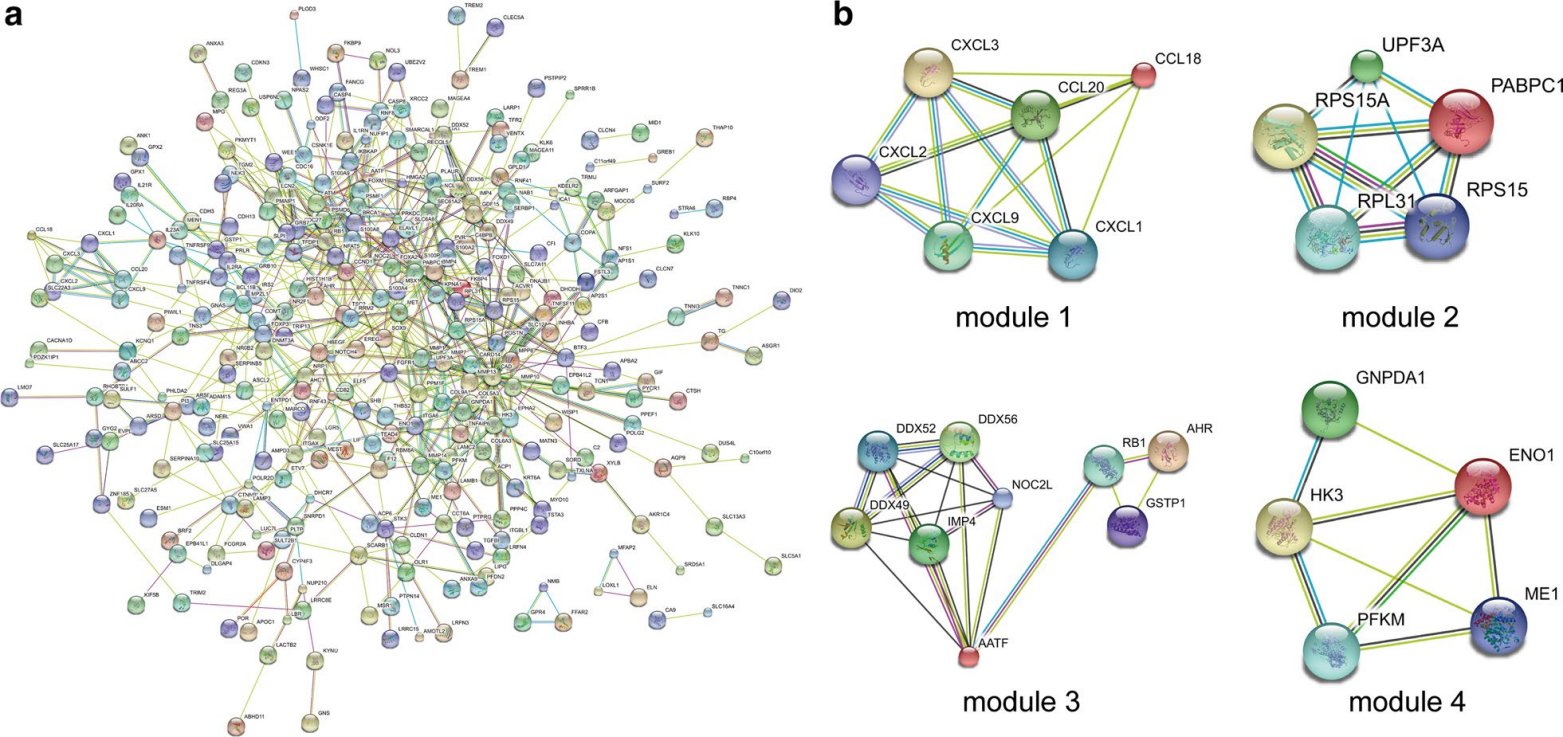
PPI network and top four modules of hypomethylation-high expression genes (**a** PPI network; **b** top module 1–4)

**Fig. 4:**
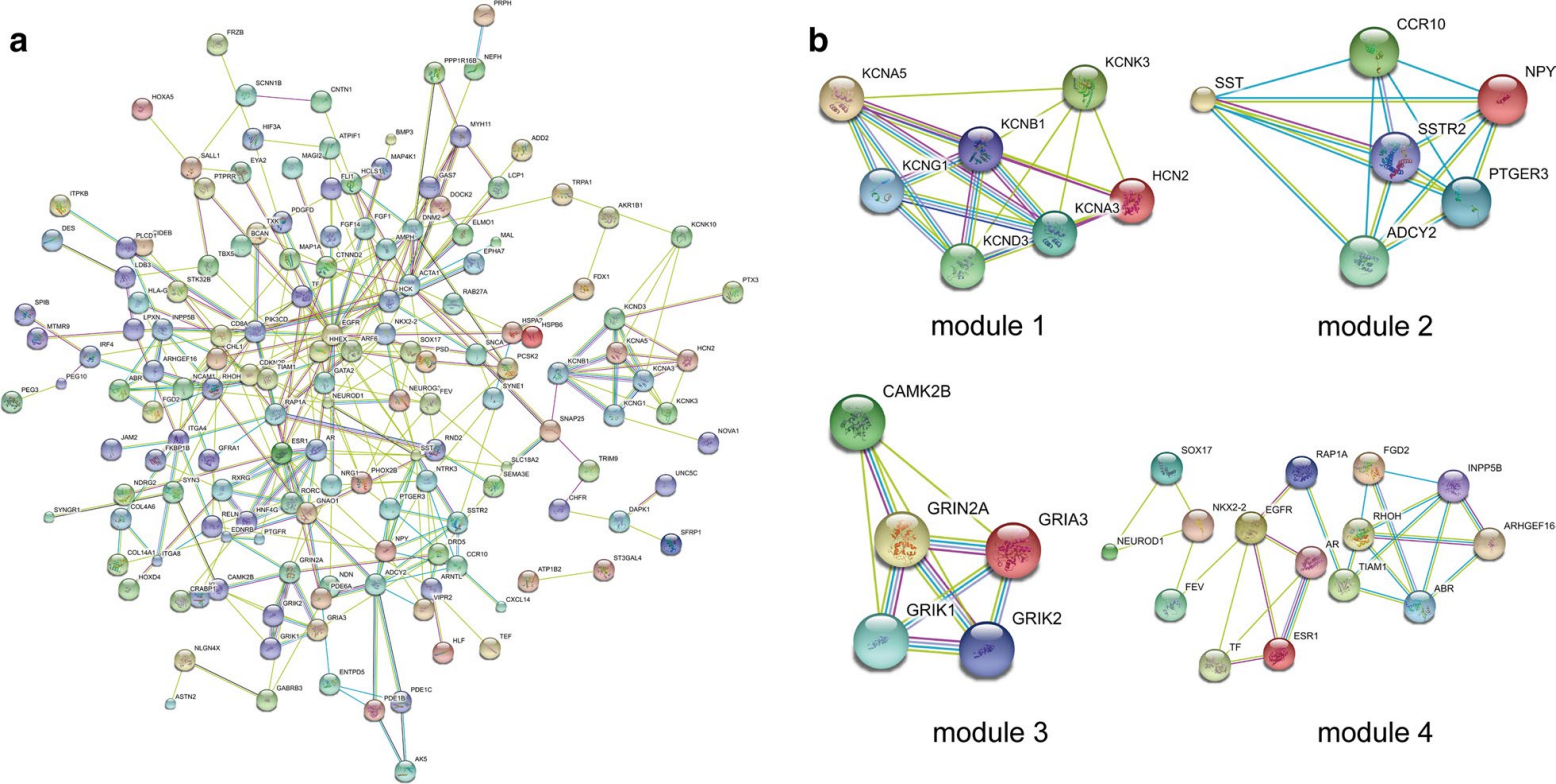
PPI network and top four modules of hypermethylation-low expression genes (**a** PPI network; **b** top module 1–4)

PPI networks were constructed on the basis of STRING database. Module analysis was conducted by MCODE in Cytoscape software. For hypomethylation-high expression genes, PPI network was shown in Fig. 3a and top four modules were displayed in Fig. 3b. Significant core modules demonstrated functions of chemokine signaling, ribosome, nucleic acid binding, carbon metabolism, cell cycle and proteasome (Table 3). Top five hub genes were CAD, CCND1, ATM, RB1 and MET. PPI network of hypermethylation-low expression genes was illustrated in Fig. 4a and top four modules were displayed in Fig. 4b. Significant key modules showed functions including synaptic transmission, gastric acid secretion, glutamatergic synapse and cell fate specification. Top five hub genes included EGFR, ACTA1, SST, ESR1 and DNM2.

**Table 3:** Module analysis of the protein–protein interaction network

Category	Module	Function description	FDR	Nodes	Genes
Hypomethylation and high expression					
	1	Chemokine signaling pathway	1.14E−10	6	CCL18, CXCL2, CXCL1, CCL20, CXCL9, CXCL3
	2	Ribosome	6.80E−04	5	RPL31, PABPC1, UPF3A, RPS15A, RPS15
	3	Nucleic acid binding	0.0195	9	DDX49, AHR, GSTP1, RB1, AATF, DDX56, NOC2L, IMP4, DDX52
	4	Carbon metabolism	3.43E−04	5	GNPDA1, PFKM, ENO1, ME1, HK3
	5	Cell cycle	3.01E−10	8	CDC16, PKMYT1, CDC27, TFDP1, SMARCAL1, ATM, RECQL5, WEE1
	6	Proteasome	0.0344	8	FGFR1, SOX9, PSMD6, FOXD1, CCND1, MSX1, PSMF1, FOXA2
Hypermethylation and low expression					
	1	Synaptic transmission	1.04E−07	7	KCND3, KCNG1, KCNB1, HCN2, KCNK3, KCNA5, KCNA3
	2	Gastric acid secretion	2.19E−04	6	SST, NPY, SSTR2, CCR10, PTGER3, ADCY2
	3	Glutamatergic synapse	1.09E−06	5	GRIK1, GRIK2, GRIA3, GRIN2A, CAMK2B
	4	Cell fate specification	9.20E−03	15	ARHGEF16, RHOH, TIAM1, ABR, RAP1A, NEUROD1, AR, ESR1, EGFR, TF, FEV, INPP5B, SOX17, FGD2, NKX2-2

### Validation of the hub genes in TCGA database

Hypomethylation-high expression hub genes and hypermethylation-low expression hub genes were then validated in another database TCGA to confirm the results. The outcome was summarized in Table 4. For most of the hub genes, the methylation and expression status were still significant altered and same with our results, which suggest the stability and reliability of the findings.

**Table 4:** Validation of the hub genes in TCGA database

Category	Hub gene	Methylation status	P value	Expression status	P value
Hypomethylation and high expression					
	CAD	Hypomethylation	5.55E−16	Up-regulated	3.88E−18
	CCND1	Hypomethylation	0.032	Up-regulated	7.26E−17
	ATM	–	–	Up-regulated	0.006
	RB1	Hypomethylation	5.27E−03	Up-regulated	1.17E−11
	DNM2	Hypomethylation	1.36E−13	Up-regulated	5.23E−08
Hypermethylation and low expression					
	EGFR	Hypermethylation	4.65E−14	Down-regulated	7.20E−21
	ACTA1	Hypermethylation	1.64E−14	–	–
	SST	Hypermethylation	9.67E−14	Down-regulated	2.22E−31
	ESR1	Hypermethylation	1.18E−14	Down-regulated	9.62E−20
	DNM2	Hypermethylation	5.43E−06	–	–

## Discussion

Elucidating the underlying mechanisms of the initiation and development of CRC would greatly benefit the diagnosis, treatment and prognosis evaluation. In this study, we identified 411 hypomethylation-high expression genes and 239 hypermethylation-low expression genes through analyzing available data of gene expression (GSE68468, GSE44076) and gene methylation (GSE29490, GSE17648) microarrays in CRC by multiple bioinformatics tools. Enrichment of these genes demonstrated certain pathways and hub genes affected by aberrant methylation, which may provide novel insights for unraveling pathogenesis of CRC.

As was suggested by DAVID analysis, hypomethylation-high expression genes in CRC were enriched in biological processes of response to wounding, inflammatory response, cell proliferation, cell adhesion and biological adhesion. Molecular function of GO analysis showed enrichment in identical protein binding, cytokine activity, endopeptidase activity, heparin binding, protein binding and bridging. It is reasonable because frequent cell proliferation and loss of cell adhesion is an apparent hallmark of cancers including CRC [13]. Chronic inflammatory and wounding conditions in the intestinal tract have also been found to increase CRC risk [14]. As main inflammatory mediators, cytokines critically determine the pro- or anti-tumorigenic signals within the environment of CRC [15]. KEGG pathway enrichment analysis suggested significant enrichment in pathways including cytokine–cytokine receptor interaction, p53 signaling and cell cycle. It was consistent with the fact that p53 signaling was frequently dysregulated in CRC [16].

PPI network of hypomethylation-high expression genes illustrated the overview of their functional connections, of which top 5 hub genes were also selected: CAD, CCND1, ATM, RB1 and MET. CAD gene encodes a trifunctional protein which is associated with the enzymatic activities of the first 3 enzymes in the 6-step pathway of pyrimidine biosynthesis [17]. Regulatory component of the cyclin D1-CDK4 (DC) complex that phosphorylates and inhibits members of the retinoblastoma (RB) protein family including RB1 and regulates the cell-cycle during G1/S transition [18]. Therefore, CCND1 and RB1 might be candidate genes affected by aberrant methylation that modulate cell cycle during CRC progression. ATM gene encodes a protein named serine/threonine protein kinase, which is a crucial cell cycle checkpoint kinase responsible for regulating a wide variety of downstream proteins including tumor suppressor protein p53 [19]. Aberrant methylation of the promoter region of ATM has been found to associate with increased radiosensitivity in human CRC cell-line HCT-116 [20]. Besides, aberrant methylation of ATM was also detected in colorectal cancers and adenomas [21, 22]. MET gene encodes a member of the receptor tyrosine kinase family of proteins which regulate various physiological processes including proliferation, morphogenesis and survival, but little is known about its role in CRC.

Module analysis of the PPI network for hypomethylation-high expression genes suggested that chemokine signaling, ribosome, nucleic acid binding, carbon metabolism, cell cycle and proteasome might be involved in CRC development. Ribosome, nucleic acid binding and cell cycle were all critical cellular processes during DNA replication and translation, which tend to be disordered in cancer. Chemokine signaling has been found to influence CRC invasion and/or metastasis via changing tumor microenvironment [23]. As an essential process for the degradation of proteins and maintenance for homeostasis in eukaryotic cells, ubiquitin proteasome system (UPS) alternations have been linked to gastrointestinal malignancies including CRC [24]. Our findings highlighted the probable importance of the regulation of these key biological behaviors by aberrantly hypomethylation in CRC, which warranted further investigations to confirm.

For hypermethylation-low expression genes in CRC, GO analysis showed that enriched biological processes were cell–cell signaling, transmission of nerve impulse, regulation of neurological system process and neuron differentiation. Recently, nerve ablation was found to delay development of precancerous lesions and inhibit tumor growth and metastasis [25], but relevant studies were still limited in CRC. The regulation of hypermethylation for nerve related genes might become promising research direction for CRC development in the future. Molecular function enrichment revealed transcription factor activity, channel activity and passive transmembrane transporter activity. KEGG analysis displayed enrichment in pathways of calcium signaling, maturity onset diabetes of the young, cell adhesion molecules (CAMs), neuroactive ligand-receptor interaction, chemokine signaling and Fc gamma R-mediated phagocytosis. These results outlined the important roles of transcription factor, cellular channel and adhesion in CRC, which were candidate study targets for their underlying mechanisms of hypermethylation and dysregulation.

After constructing PPI network for hypermethylation-low expression genes, top 5 hub genes appeared to be EGFR, ACTA1, SST, ESR1 and DNM2. As the most significant hub gene, promoter hypermethylation of EGFR gene has been proved to be related with worse survival of CRC patients who received cetuximab treatment [26]. Additionally, upregulation of EREG expression through promoter demethylation might activate the EGFR pathway during the genesis of CRC [27], which also emphasize the role of aberrant methylation in EGFR pathway. Somatostatin, encoded by SST gene, is a hormone important for regulation of the endocrine system and proliferation of both normal and tumorigenic cells [28]. Promoter hypermethylation-related decreased somatostatin production was found to promote uncontrolled cell proliferation in CRC [29], and methylation of serum SST gene might be an independent prognostic marker in CRC [30]. These results all suggested that SST gene methylation may serve as a critical regulator during CRC development. Estrogen receptor, encoded by ESR1 gene, is important for hormone binding, DNA binding and activation of transcription. And aberrant methylation of ESR1 gene in CRC has previously been discovered by several studies [31, 32]. ACTA1 gene encodes a protein belonging to actin family, which exert functions in cell motility, structure and integrity [33]. DNM2 gene encodes dynamin-2, which is subfamily of GTP-binding proteins and implicated in cell processes such as endocytosis and cell motility [34]. The roles of ACTA1 and DNM2 and their methylation status in CRC require further explorations.

Core modules within PPI network of hypermethylation-low expression genes possessed functions including synaptic transmission, gastric acid secretion, glutamatergic synapse and cell fate specification. Up to now, little is known concerning the effect of glutamatergic synapse and synaptic transmission in CRC as well as its regulation by aberrant methylation. Cell fate specification and differentiation were key procedure during cancer development [13]. According to our analysis, hypermethylation might modulate key genes responsible for cell fate determination, thus influencing the progression of CRC. It is worth noting that biological process of gastric acid secretion might also play a critical role in colorectal carcinogenesis. Gastrin has been identified as a major regulator of gastric acid secretion [35] and it also exerts effects in different tissues promoting cell division and inhibiting apoptosis [36]. Non-amidated gastrins have been proved to accelerate the development of CRC [37]. Moreover, progastrin expression level was higher in CRC than that in normal colorectal mucosa [38], and CRC patients have elevated circulating concentrations of Gamide and total gastrins [39]. The specific roles of these key modules affected by hypermethylation in the occurrence and progression of CRC still need future experiments to elucidate.

Several limitations should be acknowledged in this investigation. The clinical parameters and prognosis were not analyzed in this study due to the availability of data. Besides, the influence of aberrantly methylation on gene expression was only validated in TCGA database. Further molecular experiments are needed to better confirm the findings of the identified genes and pathways in CRC of our investigation.

## Conclusion

In summary, our study indicated a series of aberrantly methylated-differentially expressed genes and pathways in CRC by joint bioinformatics analysis of both gene expression and gene methylation microarrays, which may contribute to the finding of molecular mechanisms underlying the initiation and development of CRC. Hub genes including CAD, CCND1, ATM, RB1, MET, EGFR, ACTA1, SST, ESR1 and DNM2 might serve as aberrantly methylation-based biomarkers for precise diagnosis and treatment of CRC in the future. Compared with individual investigation, this study is possible to come up with more reliable and accurate screening results via overlapping relevant data sets. Further molecular experiments are needed to confirm the findings of the identified candidate genes in CRC.

## Abbreviations

CRC: colorectal cancer
DEG: differentially expressed gene
DMG: differentially methylated gene
GO: gene ontology
KEGG: Kyoto Encyclopedia of Genes and Genomes
PPI: protein–protein interaction
STRING: Search Tool for the Retrieval of Interacting Genes
TCGA: The Cancer Genome Atlas
BP: biological processes
MF: molecular function
CC: cell component
